# Zooming in on the intracellular microbiome composition of bacterivorous *Acanthamoeba* isolates

**DOI:** 10.1093/ismeco/ycae016

**Published:** 2024-01-23

**Authors:** Binod Rayamajhee, Mark Willcox, Savitri Sharma, Ronnie Mooney, Constantinos Petsoglou, Paul R Badenoch, Samendra Sherchan, Fiona L Henriquez, Nicole Carnt

**Affiliations:** School of Optometry and Vision Science, Faculty of Medicine and Health, UNSW, Sydney, NSW 2052, Australia; School of Optometry and Vision Science, Faculty of Medicine and Health, UNSW, Sydney, NSW 2052, Australia; Jhaveri Microbiology Centre, Prof Brien Holden Eye Research Centre, Hyderabad Eye Research Foundation, L. V. Prasad Eye Institute (LVPEI), Hyderabad, 500034, India; School of Health and Life Sciences, University of the West of Scotland, Blantyre, PA1 2BE, United Kingdom; Sydney and Sydney Eye Hospital, South-Eastern Sydney Local Health District, Sydney, NSW 2000, Australia; Save Sight Institute, University of Sydney, Sydney, NSW 2000, Australia; College of Medicine and Public Health, Flinders University, Adelaide, 5042, Australia; School of Public Health and Tropical Medicine, Tulane University, New Orleans, LA 70112, United States; School of Health and Life Sciences, University of the West of Scotland, Blantyre, PA1 2BE, United Kingdom; School of Optometry and Vision Science, Faculty of Medicine and Health, UNSW, Sydney, NSW 2052, Australia

**Keywords:** *Acanthamoeba*, eye infection, environmental predator, training ground, sympatric lifestyle

## Abstract

*Acanthamoeba*, a free-living amoeba in water and soil, is an emerging pathogen causing severe eye infection known as *Acanthamoeba* keratitis. In its natural environment, *Acanthamoeba* performs a dual function as an environmental heterotrophic predator and host for a range of microorganisms that resist digestion. Our objective was to characterize the intracellular microorganisms of phylogenetically distinct *Acanthamoeba* spp. isolated in Australia and India through directly sequencing 16S rRNA amplicons from the amoebae. The presence of intracellular bacteria was further confirmed by *in situ* hybridization and electron microscopy. Among the 51 isolates assessed, 41% harboured intracellular bacteria which were clustered into four major phyla: Pseudomonadota (previously known as Proteobacteria), Bacteroidota (previously known as Bacteroidetes), Actinomycetota (previously known as Actinobacteria), and Bacillota (previously known as Firmicutes). The linear discriminate analysis effect size analysis identified distinct microbial abundance patterns among the sample types; *Pseudomonas* species was abundant in Australian corneal isolates (*P* < 0.007), Enterobacteriales showed higher abundance in Indian corneal isolates (*P* < 0.017), and Bacteroidota was abundant in Australian water isolates (*P* < 0.019). The bacterial beta diversity of *Acanthamoeba* isolates from keratitis patients in India and Australia significantly differed (*P* < 0.05), while alpha diversity did not vary based on the country of origin or source of isolation (*P* > 0.05). More diverse intracellular bacteria were identified in water isolates as compared with clinical isolates. Confocal and electron microscopy confirmed the bacterial cells undergoing binary fission within the amoebal host, indicating the presence of viable bacteria. This study sheds light on the possibility of a sympatric lifestyle within *Acanthamoeba*, thereby emphasizing its crucial role as a bunker and carrier of potential human pathogens.

## Graphical Abstract

**Graphical Abstract:**
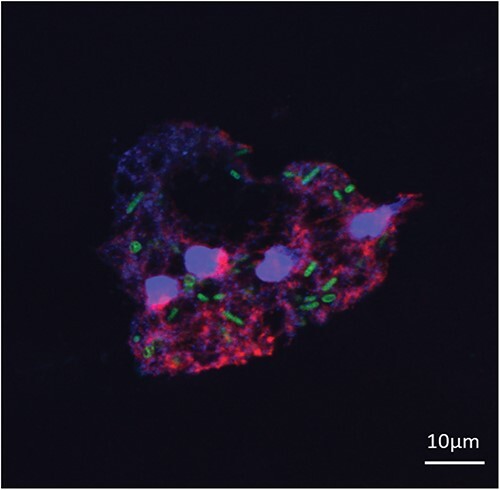

## Introduction

In recent years, *Acanthamoeba* species have become an increasingly important human pathogen, causing serious, debilitating, and sometimes deadly infections [1-4]. It can cause a rare but severe corneal infection known as *Acanthamoeba* keratitis (AK), which is extremely painful, difficult to diagnose, and treat [2]. AK can lead to vision impairment or, in severe instances, even the need for enucleation of the whole eye [5, 6]. *Acanthamoeba* can be introduced to the cornea through contaminated contact lenses, primarily due to poor hygiene practices related to contact lens usage [7]. Wearing contact lenses while showering or engaging in water recreational activities such as swimming or surfing poses a significant risk factor for AK, particularly in developed countries [8, 9]. Some of the reported outbreaks have been linked to the use of contact lens disinfecting solutions that were ineffective against *Acanthamoeba* spp. [10, 11]. In developing countries, the most frequent risk factor associated with AK is eye injury resulting from a combination of vegetative matters, dust particles, or splashing unclean water into the eyes, and trauma [12, 13].

In a remarkable dual role, *Acanthamoeba* spp. act as phagocytic predators, consuming other microbes, but also as environmental hosts for diverse microorganisms such as bacteria, fungi, and viruses [14, 15]. *Acanthamoeba* trophozoites take up microbes through phagocytosis using acanthopodia [16]. Normally, *Acanthamoeba* digest the intracellular microbes in acidic phagolysosomes [17, 18]. However, some microbes appear to be able to circumvent this and remain as viable intracellular bacteria [16, 19]. Some of these microbes can exploit amoebal cells as a natural host enhancing persistence and transmission in the environment [20, 21].

Notably, *Acanthamoeba* can package and discharge undigested bacteria such as *Vibrio cholerae* in the form of expelled food vacuoles (EFVs), which can protect the bacteria from multiple external stresses and make them more infectious both *in vitro* and *in vivo* [14]. Due to the random feeding feature of *Acanthamoeba* [22], the intracellular multi-microbial communities in the same food vacuole could serve as a “genetic melting pot” and enhance the emergence of microbes with increased abilities to endure intracellularly in amoeba as well as in cells of higher eukaryotes [16]. Such patho-adaptations in *Acanthamoeba* hosts are now broadly accepted as an environmental training ground for the evolution and transmission of potential bacterial pathogens [23].

*Acanthamoeba* spp. containing intracellular bacteria such as *Mycobacterium*, *Pseudomonas*, and *Chlamydia* have a rapid and increased cytopathic effect in a human corneal tissue model as compared with isolates devoid of intracellular bacteria [24, 25], indicating enhanced *Acanthamoeba* pathogenic potential. *Acanthamoeba* spp. that have ingested strains of *Pseudomonas aeruginosa* were more protected against disinfectants found in contact lens solutions [26]. The presence of intracellular *P. aeruginosa* was a determinant of the severity of infection in a rabbit model of AK [27]. Clinically, the presence of intracellular bacteria in corneal isolates of *Acanthamoeba* spp. was found to be associated with a tendency towards reduced initial visual acuity, longer symptom duration at presentation, and delayed diagnosis [28]. Although a retrospective study of AK versus keratitis from which *Acanthamoeba* and bacteria were cultured showed no significant differences in the disease at presentation or final outcome, this could be due to the use of broad-spectrum antimicrobials for treatment [29], and it was not certain whether the co-infecting microbes had originally been part of the *Acanthamoeba*’s microbiome. Co-infection is often observed among AK patients with multiple bacterial, viral, and fungal species [30, 31].

Understanding the types of bacteria present inside *Acanthamoeba* can provide insights into their impact on infections caused by *Acanthamoeba* spp. Therefore, this study was designed with the principal aim to investigate the composition of intracellular microbiome of *Acanthamoeba* isolates recovered from the keratitis patients, nasal mucosa, and water samples.

## Materials and methods

### *Acanthamoeba* strains, sample source, and country of origin

The source and country of origin of the *Acanthamoeba* strains assessed in this study are given in Table S1 and Fig. S1. A total of 51 isolates were included with 33 isolates from Australia (19 corneal, 9 water, and 5 nasal mucosa isolates), 13 from India (all corneal isolates), and five were ATCC strains (two isolates obtained from human corneal samples in the UK, ATCC 30873 and 30868), one isolate derived from swimming pool water in France (ATCC 30841), another isolated from cell culture in India (ATCC 30171), and one strain cultured from freshwater in the USA (ATCC 30871). Among the 51 *Acanthamoeba* isolates assessed, 28 were previously isolated and stored frozen, while the other 23 strains were isolated in this study.

### Culture and axenic maintenance of *Acanthamoeba*

All *Acanthamoeba* isolates were adapted to axenic culture and grown in peptone–yeast extract/glucose (PYG) medium (pH 6.5, 20 g of Bacto Proteose Peptone and 2 g of BD yeast extract in 950 mL of sterile water, 50 mL of 2 M D(+)glucose, 10 mL of 0.4 M MgSO_4_.7H_2_O, 10 mL of 0.005 M Fe(NH_4_)_2_(SO_4_)_2_.6H_2_O, 10 mL of 0.25 M KH_2_PO_4_ and 10.0 mL of 0.25 M Na_2_HPO_4_.7H_2_O) at 32°C. In order to avoid any potential contamination, the culture medium was substituted with freshly prepared PYG every 72 h until the trophozoites were harvested. Additionally, a separate sterile incubator, maintained at a temperature of 32°C, was used exclusively for this study. Each strain was seeded in a separate well of 24-well culture plate (Corning Incorporated, Maine, USA) with 1 mL PYG medium supplemented with 200 μL/mL penicillin–streptomycin (Thermo Fisher, USA) to kill extracellular bacteria and prevent contamination. All culture plates were incubated statically at 32°C until the trophozoites formed >90% confluent layers at the bottom of the wells. To examine the presence of bacteria in medium, aliquots (20 μL from each well) of PYG were inoculated onto trypticase soy agar (TSA; Becton, Dickinson, and Company, Sparks, MD, USA) and incubated for 48 h at 37°C. Following incubation, the growth of any bacteria on the agar plates was excluded from the study. Furthermore, we employed propidium monoazide (PMA) treatment as an additional measure to mask the DNA of non-engulfed bacteria, membrane-compromised cells, and free DNA [32]. This treatment was carried out just prior to DNA extraction, ensuring the accurate preservation of the targeted intracellular bacteria DNA.

### DNA extraction, PCR, and 18S rRNA genes sequencing of *Acanthamoeba* isolates

*Acanthamoeba* genotypes were identified by PCR followed by sequencing of 18S rRNA. Amoebal cells grown in PYG were harvested in 1 mL of 1X PBS (2.7 mM KCl, 1.4 mM NaCl, 10 mM Na_2_HPO_4_ and 1.8 mM KH_2_PO_4_, pH 6.9) and centrifuged for 10 min at 500×g and washed three times with 1X PBS to remove the medium. Nuclear DNA was extracted using DNeasy blood and tissue kit (Qiagen, GmbH, Hilden, Germany) according to the manufacturer’s instructions. DNA concentration was measured using Nano Drop UV–Vis spectrophotometer (Thermo Fisher Scientific) and dsDNA vials were stored at −20°C until further use. The PCR reaction was performed with a primer pair specific to the *Acanthamoeba* genus that comprised the forward primer JDP1 (5’-GGC CCA GAT CGT TTA CCG TGAA-3′) and the reverse primer JDP2 (5’-TCT CAC AAG CTG CTA GGG GAG TCA -3′) [33]. These primers are designed to amplify the highly variable DF3 region of the 18S rRNA i.e. *Rns* gene and generate amplicons of ~450 bp. PCR amplification was carried out as described previously [34]. Briefly, 25 μL of reaction mixture consists of 12.5 μL of DreamTaq Master Mix (DNA Polymerase, 2X DreamTaq buffer, dATP, dCTP, dGTP and dTTP: 0.4 mM each, and 4 mM MgCl_2_; Thermo Fisher Scientific), 6.5 μL of PCR water, 1 μL of each primer (10 μM) and 4 μL of DNA template with thermal cycles as follows: initial denaturation at 95°C for 5 min, followed by 35 runs of amplification (94°C for 30 s, 56°C for 30 s, and 72°C for 45 s) and a final extension at 72°C for 10 min. The PCR products were visualized in 1% agarose gel, and PCR positive amplicons were sent to the Ramaciotti Centre for Genomics (UNSW, Sydney) for Sanger sequencing with primer JDPFw (5’-GGC CCA GAT CGT TTA CCG TGAA-3′) using BigDye Terminator (V3.1) reaction mix in 3730 DNA analyser (Applied Biosystems, Massachusetts, USA). The trimmed sequence reads were subjected to a BLASTn search against the NCBI nucleotide sequences database to determine the *Acanthamoeba* genotypes. The sequences were aligned using the ClustalW algorithm, and then a phylogenetic tree was generated with the neighbour joining (NJ) approach and Bayesian approach using Kimura-2 parameters with 1000 bootstraps in MEGA-X [35].

### Genomic DNA extraction targeting intracellular bacteria in *Acanthamoeba* strains

*Acanthamoeba* isolates for strain identification and for intracellular bacteria characterization were cultured separately in axenic conditions. *Acanthamoeba* strains grown in 12-well culture plates with PYG medium containing 200 μL/mL penicillin-streptomycin were put on ice with gentle agitation to dislodge adhered trophozoites. The trophozoites were suspended in Page’s modified Neff’s amoeba saline (PAS; 1.2 g NaCl, 0.03 g CaCl_2_, 0.04 g MgSO_4_.7H_2_O, 1.36 g KH_2_PO_4_, and 1.42 g Na_2_HPO_4_ in 1 L distilled H_2_O) followed by centrifugation for 10 min at 500× g and washed three times with PAS. Amoebal cells were forced through a 29G ultrafine syringe (BD, Sparks, MD, USA) to completely lyse them. The lysate was centrifuged at 500× g for 5 min for cell pellet acquisition. Total DNA was extracted using DNeasy blood and tissue kit following manufacturer recommendations. The presence of intracellular bacteria in each *Acanthamoeba* strain was first assessed using eubacteria 16S rRNA PCR primers (341Fw and 785Rv) as described previously [36]. The positive control in the 16S rRNA PCR was DNA extracted from *Escherichia coli* ATCC 10798, while nuclease free water was used as the negative control. To further confirm the axenic culture, unused PYG medium and medium from *Acanthamoeba* culture plates were included in the PCR experiment. Genomic DNA isolated from *Acanthamoeba* isolates that tested positive for bacterial DNA in PCR assay were sent for bacterial microbiome analysis.

### 16S rRNA gene library preparation and sequencing

Bacterial 16S rRNA gene was PCR amplified targeting the V1–3 region using primer pair (27Fw, AGA GTT TGA TCA TGG CTC AG, and 519Rv, GTA TTA CCG CGG CTG CTG) with added Illumina adapter overhang nucleotide sequences [37]. Amplicon libraries were prepared and indexed using Nextera XT Index Kit. Library validation was carried out using Agilent 4200 Tape station kit on the Illumina MiSeq platform (2× 300 bp sequence mode) following the Illumina sequencing procedure for pair-end sequencing at the Ramaciotti Centre for Genomics (UNSW, Sydney). The reaction mixture for index PCR (per 25 μL reaction) consisted of 12 μL molecular grade water, 1 μL forward index primer (10 μM), 1 μL reverse index primer (10 μM), 1 μL template DNA, and 10 μL KAPA HiFi Hot Start DNA polymerase (Roche Cat No. KK2602) containing dNTPs, MgCl_2_, and stabilizers. Amplification was performed with the following thermocycler conditions: 95°C for 3 min followed by 35 cycles of 98°C for 20 s, 55°C for 10 s, 72°C for 45 s, and 72°C for 5 min, followed by holding at 4°C. The final PCR amplicons were purified and quantified, and libraries were pooled in equimolar amounts. The pooled library (10 pM) was loaded in the MiSeq Reagent Kit (Illumina Inc, San Diego, CA) and paired end sequencing (2× 300 bp) was performed. To monitor for background contamination, a negative control with no template was sequenced alongside the samples.

### Sequence processing and analysis

Analysis of the data was performed using the Windows version of Microsoft Excel 2021 (Microsoft Corporation, Washington, USA) and R software (version 4.3.0). Visuals were generated using GraphPad Prism 8.0 (GraphPad Software, San Diego, CA, USA). The sequencing quality scores of 16S rRNA genes were assessed with FastQC (www.bioinformatics.babraham.ac.uk/projects/fastqc). Raw sequences (FastQ) generated out of the Illumina MiSeq were analysed and quality filtered using Mothur [38] (version 1.43.0) platform following the Mothur MiSeq standard operating method [39]. Briefly, primer and adaptor sequences trimmed, and quality filtered sequences were examined to determine amplicon sequence variants (ASVs) with the DADA2 pipeline [40] implemented in R package dada2 v1.24.0. Forward reads with ≤5 expected errors and reverse reads with ≤10 expected errors were retained. Error-corrected reads with a minimum overlap of 20 bp and ≤ 1 mismatches in the overlap region were merged to contigs (ASVs). Chimeric contigs consisting of two partial sequences of different origin were removed with the “consensus” procedure implemented in DADA2. Remaining contigs were taxonomically classified with the IDTAXA approach [41] implemented in R package DECIPHER v2.24.0 [42] using the SILVA small subunit rRNA database (SSU, release 138) [43]. Sequences that had a classification confidence value of ≥50% were binned into ASVs list. Based on the NJ approach, a phylogenetic tree was constructed from aligned ASVs with R package DECIPHER. Vsearch (v 2.22.1) was used to identify and remove chimeric sequences. Prior to analysis, archaea, chloroplast, eukaryota-derived, and mitochondrial sequences were removed from the sequence files, as well as the ASVs that fail to classify as bacteria at the kingdom level and unclassified ASVs at the phylum level. Samples that had <1000 quality filtered read counts were not included in the analysis [44]. To provide a more accurate estimate of actual ASVs abundances, ASVs copy numbers were inferred by hidden-state prediction [45, 46]. Copy numbers were set to 1 for ASVs with Nearest Sequenced Taxon Index missing or > 2 (if any). ASV counts were normalized by dividing them by their respective copy number. The result was multiplied by a sample-specific factor (ratio of original to normalized ASV counts) to preserve the total count per sample. Counts were then rounded up to make them integers while preserving singletons. All subsequent analyses are based on copy number normalized ASV counts.

ASVs, taxonomic, sample metadata tables, in addition to the phylogenetic tree, were imported into R, and a Phyloseq object was created [47]. Phyloseq’s “plot_bar” function was used to create a bar plot of sample abundances. Rarefaction analysis was performed to estimate whether the observed sequence sampling depths had achieved a complete representation of the *Acanthamoeba* strains associated microbiome. The relative abundances of bacterial taxa were assessed between groups based on origin of country (India vs Australia) and source of isolation (clinical vs water). To aid visual representation, taxa that had a relative abundance of <1% in all samples were grouped into a category labelled as “<1% abundant taxa”. Bacterial diversity (beta and alpha) metrics were analysed using Phyloseq [47] R-package (v1.42.0). Alpha diversity within samples was evaluated using Observed ASVs, Chao1, Shannon and Simpson indexes. The beta diversity between samples was compared using principal coordinates analysis (PCoA) plots using both non-phylogenetic-based (Bray–Curtis dissimilarity index) and phylogenetic-based (weighted and unweighted UniFrac distances) metrics. A phyloseq-class object containing ASV-table plus phylogenetic tree and ASV-table were used as input for calculating the UniFrac and Bray–Curtis distance metrics, respectively. Significant differences between groups were determined using R’s wilcox.test for the Wilcoxon rank sum test (two groups) or kruskal.test for the Kruskal–Wallis test (>two groups). Permutational multivariate analysis of variance (PERMANOVA), implemented as “adonis2” function in the vegan (v2.6-4) R-package [48], was used to assess the microbiome profile (beta diversity metrics) among and within groups. When multiple comparison testing was carried out, the Benjamini–Hochberg (BH), a post hoc correction was applied to control the false-discovery rate. Adjusted *P*-values were considered significant at *P* < 0.05.

### Fluorescence *in situ* hybridization

In order to visualize the presence of intracellular bacteria in *Acanthamoeba* strains, Fluorescence *in situ* hybridization (FISH) in combination with fluorescence microscopy was performed as previously described [49]. Briefly, 1 ml amoebal cells containing >95% trophozoites were harvested from axenic cultures and washed three times with 1X Page’s saline. About 25 μL of amoebic suspension was transferred on poly-l-lysine coated slides (Thermo Scientific, Braunschweig, Germany) and left for 20 min at room temperature. The attached cells were fixed with 50 μL of 4% paraformaldehyde (buffered, pH 6.9) for 20 min at 25°C. Then the fixed cells were washed with 1X PBS, dehydrated in increasing concentration of ethanol (50, 80, and 96%), 3 min for each and air-dried before subjected to hybridization assay. Intracellular bacteria were examined by hybridization using probe EUB338, which specifically hybridized to the complementary sequence of 16S rRNA of all bacteria, as well as probe pB-914, which targets bacteria of the Enterobacteriaceae family. Additionally, probe EUK516 was used to label the 18S rRNA of trophozoites (Biomers, Ulm, Germany) (Table S2). To perform hybridization, 1 μL of each probe (50 ng/μL) was mixed with 9 μL of hybridization buffer containing 20 mM Tris–HCl (pH 7.1), 900 mM NaCl, 0.01% SDS, and 20% v/v formamide then added (30 μL/sample) to the fixed amoebal cells on slides. All slides were kept in the dark at 46°C for a minimum of 1.5 h. Subsequently, the slides were rinsed with 20 μL of pre-warmed buffer (containing 20 mM Tris/HCl at pH 7.2, 180 mM NaCl, and 0.01% SDS) at 48°C. Post-hybridization washing was performed in dark at 52°C for 20 min with 300 μL buffer on the slide. All slides were dried at room temperature and were mounted using Prolong Diamond Antifade with DAPI (Thermo Fisher Scientific) then FISH-stained slides were visualized using confocal microscope (Olympus FV1200) and images were analysed in ImageJ.

### Transmission electron microscopy

*Acanthamoeba* cells were collected from culture medium, washed with 1X PBS (three times) and pelleted by centrifugation (500× g, 5 min). The washed cell pellets were fixed in 2.5% (w/v) glutaraldehyde in 0.2 M sodium phosphate buffer at 4°C overnight. Fixed samples were rinsed with 0.1 M sodium phosphate buffer and post fixed in 1% osmium tetroxide with 1.5% potassium ferrocyanide in 0.2 M sodium buffer by using a BioWave Pro+ Microwave Tissue Processor (Ted Pella, California, USA). After rinsing with 0.1 M sodium phosphate buffer, samples were dehydrated in a graded series of ethanol (30, 50, 70, 80, 90, and 100%) followed by infiltration with resin (Procure, 812). After resin infiltration overnight, samples in resin were polymerized using an oven at 60°C for 48 h. Ultrathin sectioning of 70 nm was cut using a diamond knife (Diatome, Nidau, Switzerland) and collected onto carbon-coated copper slot transmission electron microscopy (TEM) grids. Grids were post-stained using 2% uranyl acetate and lead citrate. Two grids were collected for each sample and imaged using an ultra-high resolution scanning JEOL TEM-1400 (Tokyo, Japan) operating at 100 kV.

## Results

The electrophoresis of PCR amplicons from 21 *Acanthamoeba* isolates revealed bacterial DNA bands (Fig. S2A–C). The PYG medium was supplemented with penicillin–streptomycin, and as a result, none of the aliquots from the isolates exhibited positive culture growth on TSA, indicating the presence of intracellular bacteria in *Acanthamoeba* isolates that tested positive for 16S rRNA PCR. Out of the 51 *Acanthamoeba* strains analysed, 41.2% of the isolates tested positive for intracellular bacteria in the PCR assay, with 61.5% (8/13) of *Acanthamoeba* isolates recovered from AK patients in India, 36.9% (7/19) isolated from corneal scrapes in Australia and 55.6% (5/9) from water samples in Australia being positive for intracellular bacteria. Additionally, *Acanthamoeba culbertsoni* (ATCC 30171) harboured intracellular bacteria (Table 1).

**Table 1:** List of *Acanthamoeba* isolates positive for 16S rRNA used for profiling of intracellular bacterial microbiome composition.

S.N.	Strain lab ID	Study code	*Acanthamoeba* species, genotype	Sample source	Sample geosphere
1	L-579/20	Ac31	*Acanthamoeba polyphaga*, T4B	Human cornea	India
2	L-604/20	Ac32	*Acanthamoeba* sp., T4B		
3	L-1133/20	Ac33	*A. culbertsoni*, T4B		
4	L-1137/20	Ac34	*A. triangularis,* T4F		
5	L-1326/20	Ac36	*A. polyphaga,* T4B		
6	L-2391/20	Ac38	*A. healyi*, T12		
7	L-2482/20	Ac40	*A. culbertsoni*, T4B		
8	L-2483/20	Ac41	*A. culbertsoni*, T4B		
9	Ac-112	Ac7	*Acanthamoeba* sp., T4D	Human cornea	Australia
10	Ac-139	Ac28	*Acanthamoeba* sp., T4A		
11	Ac-98	Ac12	*Acanthamoeba* sp., T4D		
12	Ac-99	Ac13	*Acanthamoeba* sp., T4D		
13	Ac-100	Ac20	*Acanthamoeba* sp., T4A		
14	Ac-101	Ac23	*A. lenticulate*, T5		
15	Ac-102	Ac29	*Acanthamoeba* sp., T4A		
16	Ac-001 (ATCC)	Ac1	*A. culbertsoni*, T10	Cell culture	India
17	R3	Ac43	*Acanthamoeba* sp., T4F	River water	Australia
18	Ac-89	Ac44	*Acanthamoeba* sp., T4A	Water supply dam	
19	Ac-32	Ac47	*Acanthamoeba* sp., T4F		
20	Ac-059	Ac49	*Acanthamoeba* sp., T4D		
21	Ac-71	Ac51	*Acanthamoeba* sp., T4D		

### Genotypic analysis and phylogenetics of *Acanthamoeba* isolates

The partial nucleotide sequences of 18S rRNA (DF3 region) of 21 *Acanthamoeba* strains were aligned using ClustalW algorithm and compared with the NCBI database to confirm genus using BLASTn searches. The analysed sequences exhibited high similarities (>97%) with genus *Acanthamoeba*. A phylogenetic tree was constructed using the NJ method (1000 bootstraps in Kimura parameter) with reference nucleotide sequences from genotypes T1, T2, T3, T4 (A-G), T5, T6, T12, and T13 [50]. Genotype T4 accounted for the majority of the isolates (85.7%), with T12 (L-2391/20), T10 (Ac-001), and T5 (Ac-101) each represented by a single strain (Fig. S3). Among the T4 isolates (*n* = 18), four sub-clusters were identified; T4B (*n* = 6), T4D (*n* = 5), T4A (*n* = 4), and T4F (*n* = 3). Additionally, a genotype T12 strain (L-2391/20) was recovered from a keratitis patient in India [34], and a T5 strain (Ac-101) was isolated from a patient with contact lens-related keratitis in South Australia (Fig. S3).

### 16S V1–3 sequencing of *Acanthamoeba* associated intracellular bacteria

An average of 82 549 ± 30 667 reads was retained for each isolate after quality filtering (Table S3). The rarefaction curve of bacterial richness (observed ASVs) was plotted as a function of the sequencing depth indicates that all the samples had sufficient reads to capture most of the bacterial community diversity implying adequate sample coverage to proceed further (Fig. S4). The sequence file of *A. culbertsoni* (ATCC 30171) was removed from the data set due to <200 reads post quality filtering, leaving 20 samples for downstream analyses. The negative control, which was sequenced to monitor potential background contamination, was also excluded from further analysis because it had only 355 reads post quality control steps.

**Figure 1:**
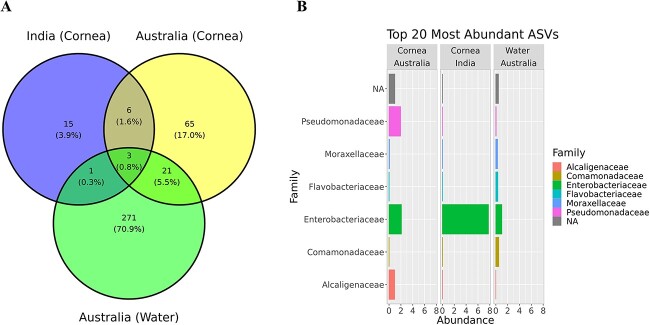
Venn-diagram showing unique and shared ASVs (relative abundance >0) among different *Acanthamoeba* groups as per source of isolation and origin of country (**A**). The top 20 most abundant ASVs clustered into six different bacterial families cross all *Acanthamoeba* isolates as per source of isolation and origin of country (**B**).

A total of 382 unique ASVs were obtained from 20 *Acanthamoeba* isolates sequenced for the 16S V1-V3 rRNA. Of these, 271 (70.9%) unique ASVs belonged to water isolates, 65 (17%) to corneal isolates of *Acanthamoeba* spp. recovered in Australia, and 15 (3.9%) were attributed to Indian keratitis strains (Fig. 1A and Fig. S5). The total ASVs clustered into six different phyla and the majority of ASVs (93.5%) belonged to Gram-negative bacteria with over 82% AVSs belonging to Pseudomonadota followed by Bacteroidota (10.7%). The top 20 most abundant ASVs belonged to six different bacterial families (Fig. 1B and Fig. S6). An average of five ASVs were observed in each *Acanthamoeba* obtained from the corneal samples and 11 ASVs in water strains isolated in Australia and there was an average of 6 ASVs per *Acanthamoeba* cultured from keratitis patients in India (excluding <1% of total ASVs counted).

### Bacterial microbiome diversity and composition

**Figure 2:**
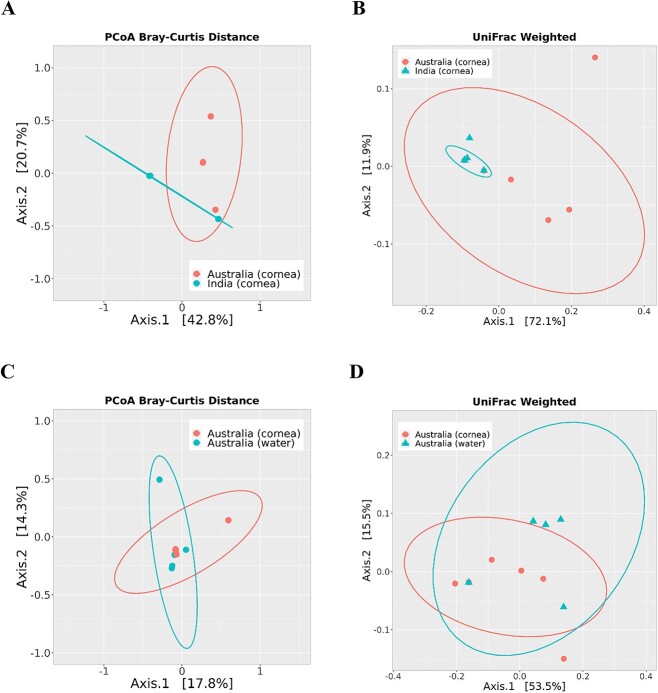
Beta diversity of bacterial microbiome composition in *Acanthamoeba* corneal isolates was compared between the countries of origin, India (*n* = 8) and Australia (*n* = 7), and also within Australian isolates based on their sources: corneal (*n* = 7) and water isolates (*n* = 5). Two-dimensional PCoA plots comparing Bray–Curtis dissimilarity index (**A**) and weighted UniFrac distance metric (**B**) show significant differences (*P* < 0.05) between Indian and Australian corneal isolates of *Acanthamoeba* spp., but no significant differences (*P* > 0.05) between Australian water and corneal isolates (**C**, **D**). The axes represent the first two principal coordinates of the PCoA plot, with each point on the plot representing the bacterial microbiome of an individual *Acanthamoeba* strain. The ASVs data were transformed to relative abundance before plotting to account for differences in sequencing depth and some of the sample points are overlapped on the plots due to the very similar bacterial microbiome composition.

The isolates were categorized as India (cornea), Australia (cornea), and Australia (water) for comparison of microbiome diversity. Two-dimensional PCoA plots calculated at the ASVs level using weighted UniFrac distance metric and Bray–Curtis dissimilarity index revealed significant differences in bacterial microbiome composition between *Acanthamoeba* isolates obtained from keratitis patients in India and Australia (*P* < 0.05) (Fig. 2A–B). The bacterial microbiome composition (beta diversity) of *Acanthamoeba* isolates was non-significant between the corneal and water strains isolated in Australia (all *P*-values >0.05) (Fig. 2C–D). The beta diversity ordination, based on PERMANOVA test of Jaccard distance index, showed similar results, indicating that bacterial species diversity between *Acanthamoeba* isolates varied according to country of origin rather than source of isolation (Figs S7 and S8).

**Figure 3:**
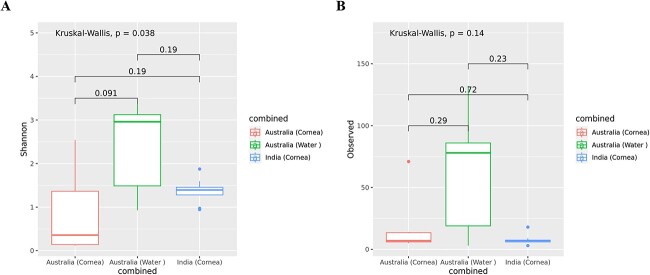
Alpha diversity of bacterial microbiome composition of *Acanthamoeba* strains by group; (**A)** Shannon index, and (**B)** number of observed ASVs. A global Kruskal–Wallis test was used to perform statistical analysis among the three groups, whereas a Wilcoxon rank sum test was performed between the two groups. *Acanthamoeba* isolates; Australia water (*n* = 5), Australia cornea (*n* = 7), and India cornea (*n* = 8). The boxplots show the smallest and largest values (the 25th and 75th quartiles), the median, and outliers.

Similarly, we found a significant difference in bacterial diversity as measured by Shannon index (*P* < 0.05, the diversity of species in a community) between the three groups of *Acanthamoeba* isolates that were obtained from cornea and water samples in Australia and the corneas of AK patients in India. However, the Wilcoxon rank sum test between the two groups showed no significant differences in alpha diversity as measured by Shannon index and richness (number of observed ASVs) (Fig. 3). Additionally, the alpha diversity measures were non-significant (*P* > 0.05) with Chao1 (species richness estimator), and Simpson (evenness) index. These results indicate no statistically significant distinctions in alpha diversity measures between the two groups of *Acanthamoeba* isolates based on their source of isolation and country of origin.

**Figure 4:**
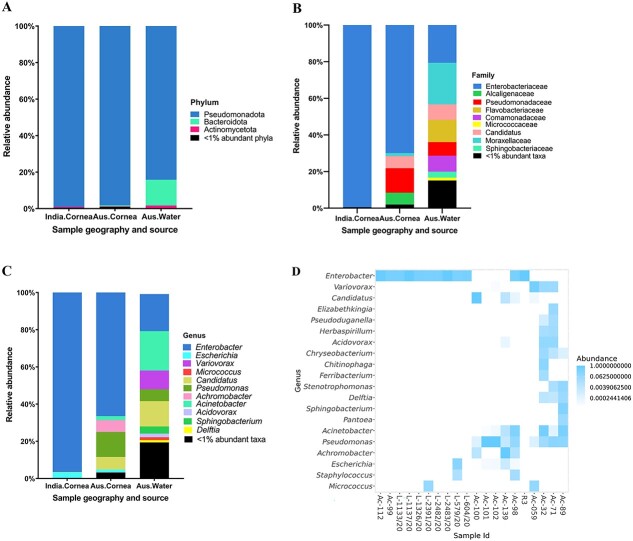
Intracellular bacterial microbiome composition of *Acanthamoeba* isolates by groups; Indian corneal isolates, Australian corneal, and water isolates. Stacked bar plots visually represent the average relative abundance (%) of 16S V1–3 rRNA gene sequences assigned to bacterial phyla (**A**), families (**B**), and genera (**C**). For visualization, taxa with <1% relative abundance have been grouped together. In cases where the genus level classification was not possible, a higher taxonomic level is mentioned and “*Candidatus*” was mentioned for *Candidatus* Jidaibacter acanthamoeba. (**D**) Heatmap representing the top 20 most abundant ASVs (log10). ASVs (genus level) are shown in y-axis and x-axis represents individual samples included for intracellular microbiome profiling of *Acanthamoeba* isolates targeting 16S rRNA, V1–3 (refer Table S1 for details of *Acanthamoeba* isolates). White cells correspond no ASVs detected. For visualization, “*Candidatus*” was labelled for *Candidatus* Jidaibacter acanthamoeba in B, C, and D.

Identifying the types of intracellular bacteria hosted by *Acanthamoeba*, particularly those recovered from clinical specimens such as corneal scrapings in keratitis cases, is important for enhancing accurate differential diagnostics and prognostic evaluations of AK. The total of 382 ASVs from 20 *Acanthamoeba* isolates were taxonomically classified into four major bacterial phyla: Pseudomonadota, Bacteroidota, Actinomycetota, and Bacillota. The linear discriminate analysis of effect size (LEfSe) was performed using LEfSe software [51]. In Australian corneal isolates, the genus *Pseudomonas* was significantly more abundant with an effect size of 5.36 (*P* < 0.007) while the order Enterobacteriales was more abundant in Indian corneal isolates, with an effect size of 5.79 (*P* < 0.017). Notably, Australian water isolates had a higher abundance of Burkholderiales and the phylum Bacteroidota with effect sizes of 5.21 (*P* < 0.011) and 5.1 (*P* < 0.019), respectively. The relative abundance of the phylum Pseudomonadota, present in all 20 isolates, was generally higher in Indian isolates (mean relative abundance = 99%) compared with Australian corneal (98%), and water isolates (84%). Overall, Pseudomonadota was the major phylum accounting for ≥84% in all three groups and Bacteroidota accounted 14% in Australian water isolates (Fig. 4A–C). At the genus level, the mean relative abundance of *Enterobacter* was relatively high in Indian clinical isolates (96%) compared with Australian corneal (67%) and water isolates (20%). *Escherichia* was only detected in corneal samples (2.5% in India and 1.5% in Australia), while *Micrococcus* accounted for 0.25% in Indian corneal strains and 1.5% in Australian water isolates. In contrast, *Pseudomonas* was relatively more abundant in Australian corneal isolates (13.2%) compared with water isolates (6%) and it was not detected in any Indian isolates. Likewise, bacterial endosymbiont *Candidatus* Jidaibacter acanthamoeba was only detected in Australian corneal (6.7%), and water isolates (13.6%) and similar observations were made for *Acinetobacter* spp. (2% and 21%, respectively). The genera *Variovorax* (10%), *Acidovorax* (2%), *Sphingobacterium* (3.8%), and *Delftia* (1.3%) were only detected in water isolates and *Achromobacter* (6.4%) was exclusively present in Australian corneal samples (Fig. 4D, and Figs S6 and S9).

### Comparison of intracellular bacteria diversity between stock and recent isolates

**Figure 5:**
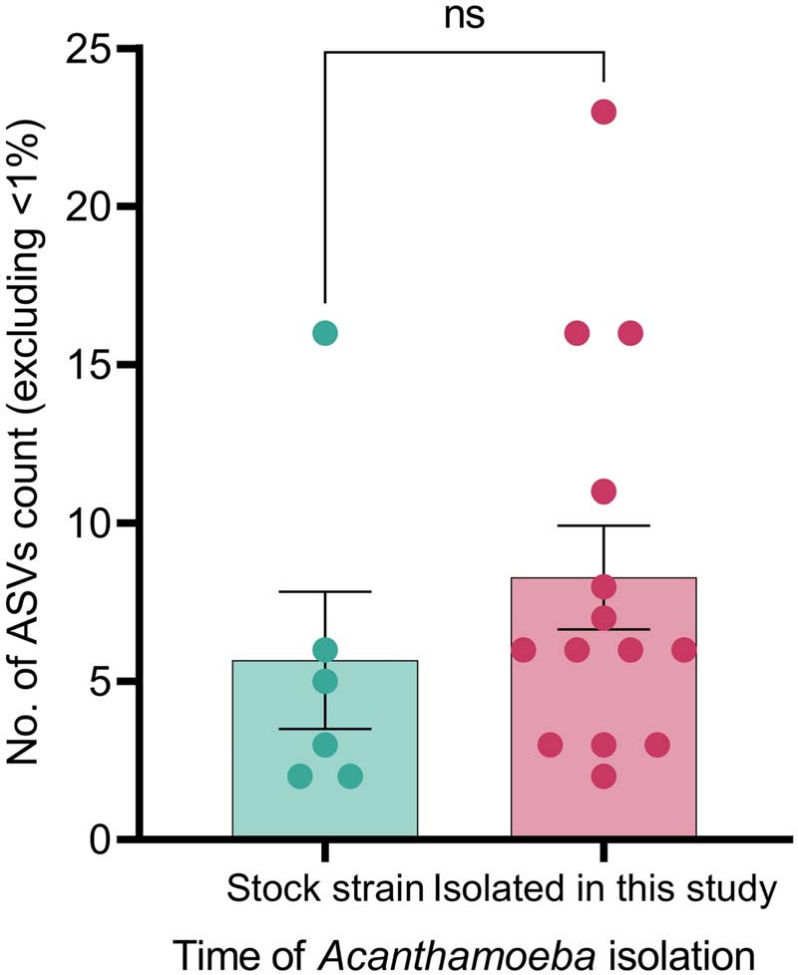
Bar plot showing the relative count of ASVs in recent and stock isolates. ASVs with <1% of the total sequence count of each isolate were excluded for calculation and visualization. Unpaired t-test was used to compare the counts between two groups.

*Acanthamoeba* strains were categorized into two groups: “stock isolates”, retrieved from our lab’s culture collection, and “recent isolates”, obtained in the current study to investigate intracellular bacterial diversity between older and newer isolates. The mean relative count of ASVs (excluding <1%) among recent isolates (8.3 ± 6.1) was insignificantly (*P* = 0.3) higher compared with stock isolates (5.6 ± 5.3) (Fig. 5). The weighted UniFrac distance and Bray–Curtis dissimilarity index showed no significant different in beta diversity metric at ASVs level (*P* > 0.05) between stock and recent isolates. The beta diversity ordination, based on the Jaccard distance index, yielded similar results, indicating no significant difference (*P* = 0.36) in bacterial ASVs diversity between stock and recent isolates (Fig. S10). Similar observations were made for alpha diversity between stock and recent isolates as measured by Shannon index, i.e. diversity of species (*P* = 0.1) and observed ASVs richness (*P* = 0.4) (Fig. S11).

### Confirmation of bacterial cells within *Acanthamoeba* trophozoites by FISH

**Figure 6:**
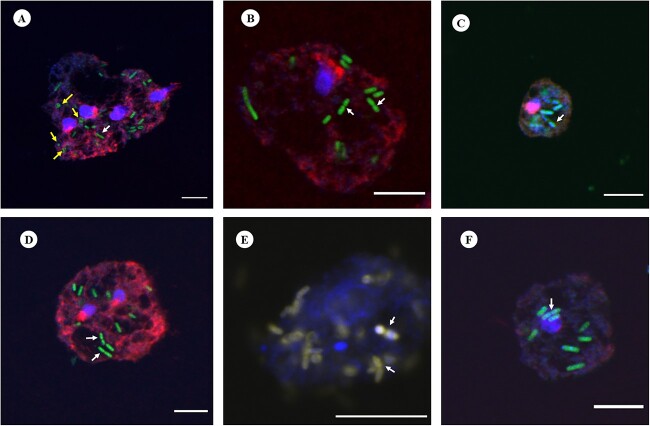
Representative FISH micrographs showing the presence of intracellular bacteria in *Acanthamoeba* trophozoites investigated in this study. Probes EUK516 conjugated with Cy5, targeting *Acanthamoeba*, and EUB conjugated with Cy3, targeting most of bacterial strains were used for all *Acanthamoeba* strains positive for bacterial 16S rRNA. DAPI was used in mounting medium when visualized by a fluorescence microscope. Probe pB-914 labelled with 6-FAM was used for isolates containing high abundance of bacteria belong to Enterobacteriaceae family. (**A**) Rod shaped bacteria were observed throughout the cytoplasm of *Acanthamoeba* trophozoites (Indian corneal isolate) and a few cocci bacteria were also observed (yellow arrows). The white arrow represents bacterium cell undergoing binary fission. (**B**) Bacteria showing binary fission (white arrows) in vacuole like structure of *Acanthamoeba* recovered from water sample (R3). (**C** and **D**) Corneal isolates of *Acanthamoeba* spp. (Ac-112 and L-579/20, respectively) with intracellular bacteria. (**E**) Intracellular bacteria labelled with probes EUB and pB-914 simultaneously in *Acanthamoeba* sp. isolated from an AK patient. (**F**) Clinical (Ac-102) isolate of *Acanthamoeba* trophozoite depicting rod shaped intracellular bacteria. Indicators: white arrow, bacterial cell undergoing binary fission; yellow arrow: cocci shaped bacteria. Scale bar in each panel represents 10 μm.

Among the *Acanthamoeba* isolates positive for intracellular bacteria, a significant proportion exhibited the presence of bacteria belonging to the Enterobacteriaceae family. To further confirm the intracellular presence of bacterial cells within the amoebal host, hybridization reactions were performed using both the universal bacterial probe EUB338 and the Enterobacteriaceae family-specific probe pB-914. Bacterial probes showed positive hybridization signals, confirming the successful binding of the probes to bacterial target sequences and bacterial cells were stained with dyes conjugated with probes (Fig. 6). Bacterial cells were found to be distributed throughout the cytoplasm of the *Acanthamoeba* host, demonstrating their presence across the entire population of amoebal cells. In addition, we observed a few bacterial cells replicating by binary fission in vacuole like structures of trophozoites (Fig. 6B–D). Furthermore, by employing simultaneous hybridization with a specific probe for Enterobacteriaceae and a universal bacterial probe labelled with distinct dyes, the presence of bacterial cells inside amoebic trophozoites was observed (Fig. 6E). For the double probes’ assays, signal intensities were almost equivalent for hybridization buffer containing 10–25% formamide.

### Ultrastructure of bacterial cells within *Acanthamoeba* host

**Figure 7:**
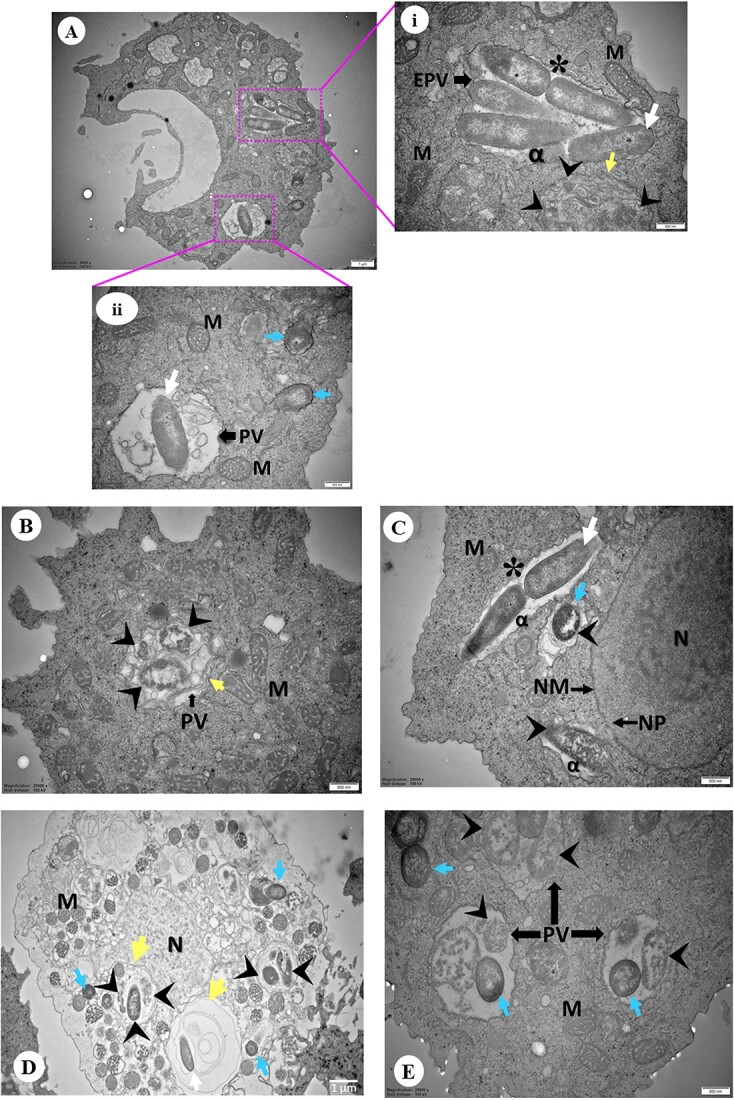
Representative images of TEM showing *Acanthamoeba* isolates containing intracellular bacteria. (**A**) Overview of an *Acanthamoeba* trophozoite (Indian corneal isolate) harbouring intracellular bacteria. (**A.i-ii**) Higher magnification showing rod (white arrow) and cocci (blue arrow) shaped bacteria inside early phagocytic (**i**) or PV (**ii**), and bacterial cells were also observed in trophozoite cytoplasm (ii). A bacterium undergoing binary fission (asterisk) and digested bacteria (arrowhead) appear disintegrated surrounded by multiple layers (yellow arrow) (ii). (**B**) Engulfed bacteria appeared disintegrated and digested inside PV surrounded by multiple layers (Australian water isolate). (**C**) Rod and spherical shaped bacterial cells close to host NM appears enclosed by double-membranous vacuole and disintegrated (arrowhead). And a bacterial cell is undergoing binary fission (Australian corneal isolate). (**D**) Engulfed bacteria appeared disintegrated and digested inside PV close to host NM. Both digested and undigested bacteria in the same PV consisting multiple layers of membrane. (**E**) Digested and undigested cocci bacteria in the same PV. Symbols = EPV: early phagocytic vacuole; PV: phagocytic vacuole; M: mitochondria; N: nucleus; NM: nuclear membrane; NP: nuclear pore; DV: digestive vacuole; CV: contractile vacuole; white arrow: rod bacteria; blue arrow: spherical bacteria; arrowhead: digested bacteria; yellow arrow: surrounded by multiple layers; asterisk (^*^): binary fission; alpha (α): electron translucent space. The lengths of bars in the bottom right corner of each image represent 500 nm except A (1 μm), and D (1 μm).

TEM was used to further investigate the intracellular niche and ultrastructure of bacterial cells residing within their amoebal host. For this analysis, one representative isolate of each sample category (Indian corneal isolates, Australian corneal and water isolates) was selected. By TEM, it is observed that bacteria were mostly pleomorphic rod-shaped, but some cocci were also found which were surrounded by electron-translucent regions of variable sizes (Fig. 7). Most of the bacterial cells were enclosed in phagosomes, which are the early phagocytic vacuoles (EPVs), while some were non-membrane bound and distributed randomly in the host cytoplasm. No intranuclear stage was detected; however, a small number of cells were observed in proximity to the nuclear membrane (NM) (Fig. 7C–D). Distinct structural alterations were observed in the amoebal mitochondria, characterized by enlargement and the accumulation of dense deposits. In some cases, a relatively large number of mitochondrial cells were observed surrounding the phagocytic vacuole (PV) containing ingested bacteria (Fig. 7B).

Transverse bacterial cell division through binary fission was observed within the translucent regions and phagosome (Fig. 7A.i and C), but no instances of division were noted within the mature phagolysosome. The vegetative trophozoites displayed PVs of varying sizes, with some being large enough to contain more than five ingested bacteria (Fig. 7E). A distinct phagosomal membrane was evident, encapsulating the engulfed bacteria (Fig. 7B and D). It was intriguing to observe undigested and digested bacteria within the same PV appeared as intact and disintegrated with granules, respectively (Fig. 7D–E). In the *Acanthamoeba* cytoplasm, a few multi-layered membrane-bound compartments were observed, containing ingested bacteria (Fig. 7B and D).

## Discussion

To our knowledge, this is the first study to profile complete intracellular bacterial microbiomes of *Acanthamoeba* strains isolated from different geographies and sample sites. Among 51 *Acanthamoeba* isolates examined in this study, 41% possessed intracellular bacteria similar to the 46% of *Acanthamoeba* spp. isolated from keratitis patients and air-conditioners possessing endocytobiotic bacteria in a study from Malaysia [52], but slightly less compared with previous studies of corneal or contact lens isolates from Iran (53%) and the USA (59%) [28, 53]. In a systematic review conducted in 2021 [54], a wide variation was observed in the proportion of *Acanthamoeba* spp. with reported intracellular microbes, ranging from 6 to 100%. Interestingly, among the studies included in that review, approximately 23% observed the presence of more than one intracellular microbe within the same *Acanthamoeba* isolate but none of the studies had utilized metagenomic approaches to comprehensively profile the intracellular microbiome. We found 55.6% of *Acanthamoeba* isolates obtained from water samples contained intracellular bacteria. Other studies have reported that 29% of *Acanthamoeba* spp. obtained from household tap water in Korea hosted bacterial endosymbionts and 12% of environmental *Acanthamoeba* isolates exhibited the presence of intracellular bacteria in Japan [55, 56]. In the current study, we maintained axenic amoebal growth in PYG medium by adding antibiotic supplements. To preserve the integrity of the *Acanthamoeba* microbiota, we also used PMA treatment to specifically inhibit the DNA of both non-internalized bacteria and free DNA, ensuring that no alterations occurred in the amoebal intracellular microbiota.

It is important to note that among the *Acanthamoeba* isolates assessed in this study, 28 (54.9%) were isolated in the past, and only 25% of them had intracellular bacteria whereas the incidence of intracellular bacteria was 60.9% among recent isolates. The stock isolates were maintained in a culture collection, and it is possible that they may have lost intracellular bacteria since the initial isolation of those *Acanthamoeba* strains [55]. In addition, the absolute abundance of intracellular bacteria within the amoebal host may change over time, so the bacterial species detected among old isolates now might differ from the original ones. Since this study hasn’t examined the stability of these bacterial species within the *Acanthamoeba* host, future studies are anticipated to determine whether intracellular bacteria are passed on during the replication of the amoebal host. However, the mean ASV count of recent and stock isolates assessed in the current study did not show a significant difference. Similarly, both beta and alpha diversity metrics for these two groups were not significantly different, suggesting that amoeba-resisting bacteria may persist silently within the amoebal host for an extended period. In a recent preprint, Issam et al. reported that they have successfully revived a 600-year old *Acanthamoeba castellanii* strain Namur, along with its Rickettsial endosymbiont *Coprolita marseillensis*, indicating that *Acanthamoeba* can survive for centuries while protecting its intracellular symbiont [57].

The voracious feeding feature of *Acanthamoeba* spp. leads to the coexistence of sympatric bacteria within the same isolate, creating a sort of “microbial village” [28, 54, 58, 59]. While these bacterial endosymbionts may not have the capability to directly cause infectious keratitis, their presence within a compromised cornea can introduce proinflammatory bacterial components. This, in turn, can intensify corneal inflammation and potentially worsen the progression and outcome of corneal infection [28]. This may also be related to the increasing incidence of coinfections in AK with bacterial, fungal, and viral strains in the form of a superinfection [60-62]. According to a retrospective study conducted in the USA using corneal scrape cultures [62], co-infection rates among AK cases were 23.6% with bacteria, 7.3% with fungi, and 4.5% with herpes simplex virus. Similarly, in a recent study conducted in South India [31], over 50% of AK patients were found to have coinfections with various microbes, including *Fusarium* spp., *Aspergillus* spp., *Pseudomonas* spp., *Stenotrophomonas* spp., *Streptococcus* spp., among others. The wide array of organisms involved in coinfections suggests that *Acanthamoeba* interactions with other organisms are likely more prevalent than currently acknowledged. Intracellular bacteria found in *Acanthamoeba* can exacerbate corneal epithelial damage as has been observed in a clinical study and a cell model [28]. Both in patients with keratitis and experimental studies, the presence of intracellular bacteria in *Acanthamoeba* is often associated with increased stromal infiltrates, epithelial defects, hypopyon, longer symptom duration, and delayed time to diagnosis, potentially resulting in poor visual outcomes [28, 34, 63]. Hence, it is imperative to accurately identify the entirety of intracellular microbes residing within the keratitis-causing amoebal host.

This study identified a total of 382 ASVs from the 20 *Acanthamoeba* samples, which were clustered into four major phyla: Pseudomonadota, Bacteroidota, Actinomycetota, and Bacillota. The dominant phylum was Pseudomonadota (present in all 20 isolates), representing at least 98% in clinical and 84% in water isolates, indicating *Acanthamoeba* harbours primarily Gram-negative bacteria. Similarly, another study identified 730 ASVs from 39 samples of social amoebae such as *Dictyostelium*, *Polysphondylium*, *Heterostelium*, and *Cavenderia*, with the taxonomy clustering into six phyla, where Pseudomonadota was the dominant phylum [64]. However, the study found a distinct bacterial microbiome in amoebae compared with the microbiomes present in their soil habitat [64]. Our study, similar to previous findings [65, 66], demonstrates a higher prevalence of Gram-negative bacteria in all isolates, suggesting a preference of *Acanthamoeba* spp. for Gram-negative bacteria. The genome of *Acanthamoeba* encodes two peptidoglycan binding proteins and six members of the lipopolysaccharide-binding protein family, which potentially contribute to selective feeding behaviours [67]. Further molecular studies are required to advance our understanding of *Acanthamoeba*’s prey preference.

We found a greater abundance of bacterial diversity at both the family and genus levels in water strains compared with corneal isolates. There were 9 ASVs common to Australian and Indian keratitis isolates, 65 unique ASVs in Australian, and 15 unique ASVs in Indian keratitis isolates, and there were significant differences in bacterial microbiome composition between *Acanthamoeba* isolates obtained from keratitis patients in India and Australia. Interestingly, the microbiome of the Australian keratitis and water isolates did not significantly vary in its beta and alpha diversities. These similarities and differences indicate that the microbiome of keratitis isolates may be derived from sources such as water where *Acanthamoeba* commonly live, rather than there being a unique microbiome associated with keratitis. This is supported by the finding that Australian keratitis isolates more commonly contained *Pseudomonas* spp. whereas the Indian keratitis isolates more commonly contained Enterobacteriales. Environmental factors can affect the amoebal minimicrobiome. Water, with its inherent diversity, provides a vast range of microhabitats that facilitate the existence of various bacterial species. This diversity may, in turn, contribute to the uptake of a broad spectrum of bacteria by voracious *Acanthamoeba* spp. A recent study has found that *Acanthamoeba* occurrence in coastal lagoon waterways was positively correlated with cyanobacteria, *Pseudomonas* spp., *Candidatus Planktoluna*, and marine bacteria of the Actinomycetota phylum [68]. This suggests that bacterivorous *Acanthamoeba* can interact with multiple bacterial species in water habitats which may directly impact its intracellular residents. Further studies are warranted to investigate whether physiochemical parameters of water influence the microbial prey grazing ability of *Acanthamoeba* in water ecosystems. The normal human ocular surface microbiota contains *Staphylococcus* spp., *Pseudomonas sp., Enterobacter sp., E. coli*, and *Acinetobacter sp.* [69, 70], so members of these genera have the potential to be acquired by corneal isolates of *Acanthamoeba* during infection. Additionally, the *Acanthamoeba* microbiome may originate from the external environment before colonizing human eye. In a recent study examining the intracellular microbiome of five keratitis isolates and two ATCC strains, bacteria belonging to the orders Clostridiales and Bacteroidales were prevalent across all isolates. Furthermore, the study identified an association between the types of intracellular bacteria and the progression of AK, with *Blautia producta* showing a positive correlation [71], which aligns with findings reported from the USA [28].

In the current study, a significant difference in bacterial beta diversity among *Acanthamoeba* isolates was observed based on their country of origin. However, no significant differences were observed in alpha diversity measures between *Acanthamoeba* isolates in terms of both country of origin and source of isolation. Consistent with our findings, there were no significant differences in alpha diversity among soil amoebae groups, while beta diversity was contingent upon the species of amoeba [64]. Similarly, no significant differences in the diversity and richness of free-living amoeba bacterial microbiomes were observed based on the source of isolation from which amoebae were isolated [72]. Further investigation, incorporating a larger sample size from various sampling locations and sources, along with multiple replicates per site, is essential to elucidate the influence of bacterial, environmental, and host factors on the formation of the microbiome in pathogenic *Acanthamoeba* spp.

## Conclusion

This work represents the first comprehensive study into the bacterial microbiome of *Acanthamoeba* spp., encompassing isolates from both keratitis patients and water sources recovered in India and Australia. Among the 51 *Acanthamoeba* spp. analysed in this study, 41% were found to host intracellular bacteria, including some potential human pathogens such as *Pseudomonas* spp., *Acinetobacter* spp., *Enterobacter* spp., and *Achromobacter* spp. Significant differences were observed in the bacterial microbiome composition of *Acanthamoeba* spp. between samples obtained from keratitis patients in India and Australia. Water isolates were found to harbour a relatively higher number of intracellular bacteria compared with clinical isolates. Given the increasing incidence of coinfections in AK patients with severe outcomes, it is crucial to identify the microbiome harboured by *Acanthamoeba* spp. in order to enhance our understanding for more accurate differential diagnostics and prognostic evaluations of *Acanthamoeba* related infections. Further studies on the role of dominant bacteria on the *Acanthamoeba* microbiome could provide valuable insights into the intricate dynamics of microbe–microbe interactions during the course of infection. This study improves our understanding of the potential existence of a sympatric lifestyle in *Acanthamoeba*, thereby emphasizing its crucial role as a carrier of intracellular microfauna. These findings open up numerous questions for future research on the impact of host and environmental factors on amoebal intracellular microbiome formation and the intricate mechanisms of host–microbe interactions.

## Supplementary Material

Table_S1_ISMECOMMUN-D-23-00038R1_ycae016

Supplemental_materials_ISMECOMMUN-D-23-00038R1_ycae016

## Data Availability

The assigned GenBank accession number of the nucleotide sequence of 21 *Acanthamoeba* isolates used for microbiome analysis ranged from OK042095 to OK042105, OQ940657 to OQ940665, OQ158989, KC438381, OQ941630, and AF019067. All the raw sequence files of microbiome 16S rRNA sequencing have been deposited in the NCBI Sequence Read Archive (SRA) under the BioProject accession PRJNA963215.
