# Supplementary material for: Zooming in on the intracellular microbiome composition of bacterivorous Acanthamoeba isolates
Source: ISME Commun. 2024 Jan 23;4(1):ycae016. doi: 10.1093/ismeco/ycae016 (PMC10945361; doi:10.1093/ismeco/ycae016)
Supplement: Supplemental_materials_ISMECOMMUN-D-23-00038R1_ycae016 [file supplemental_materials_ismecommun-d-23-00038r1_ycae016.docx]

**Supplementary Materials**

**Table S2:** FISH probe bases used in this study

| **S.N.** | **Probe** | **Specificity** | **Sequence (5’ to 3’)** | **rRNA position** | **Modification at 5′** |
| --- | --- | --- | --- | --- | --- |
|  | EUK516 | Eukaryota | ACCAGACTTGCCCTCC | 502-517 (18S) | Cy5 |
|  | EUB338 | Most bacteria | GCTGCCTCCCGTAGGAGT | 338-355 (16S) | Cy3 |
|  | pB-914 | Enterobacteriaceae | CTCTTTGGTCTTGCGACG | 183-200 (16S) | 6-FAM |


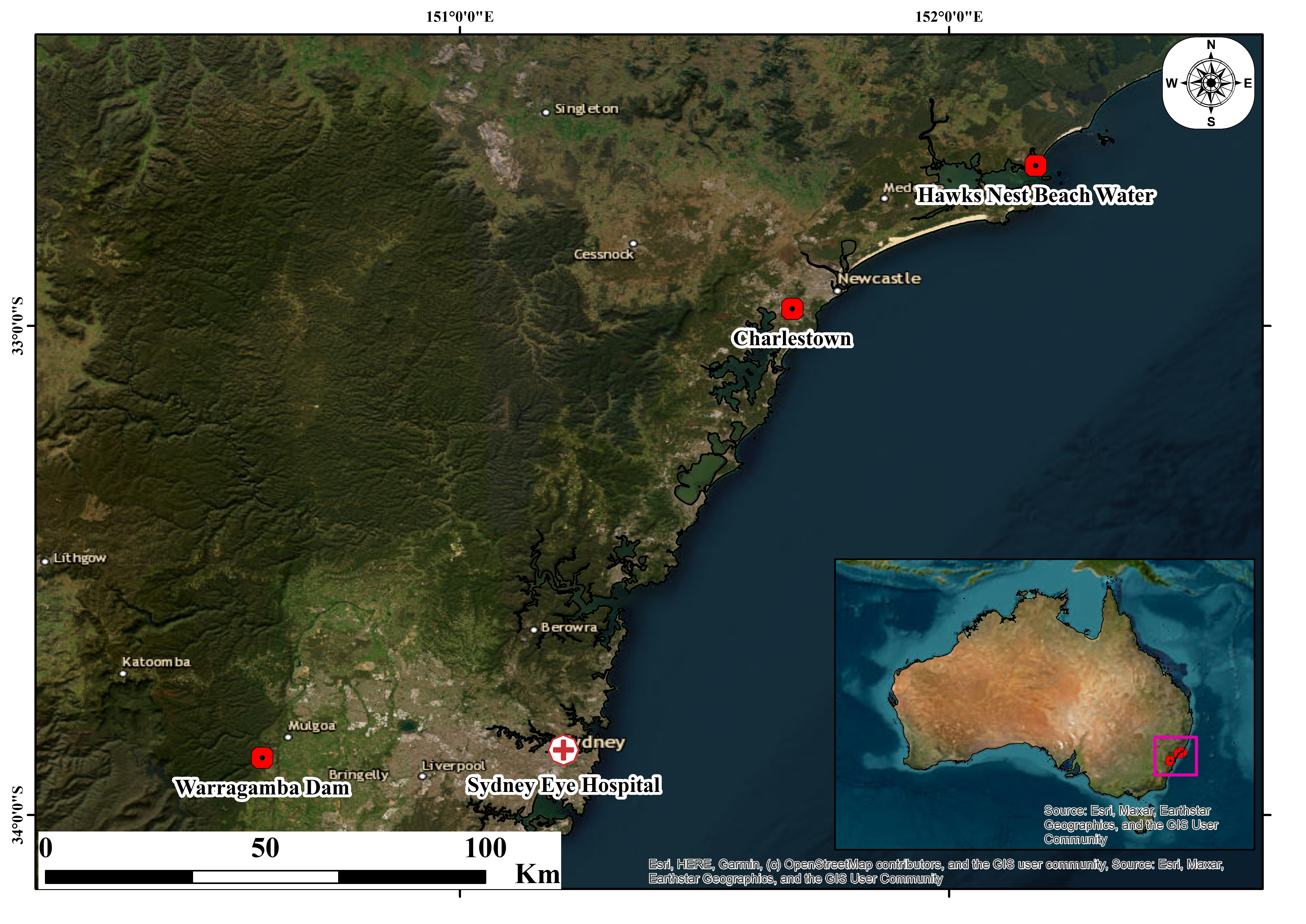


**Figure S1:** Map showing Hawks Nest Beach Water and Warragamba Dam from where water samples were collected. The map was created using ArcGIS (Esri, GIS, California, USA).

| **A.** | **B.** | **C.** |
| --- | --- | --- |
| 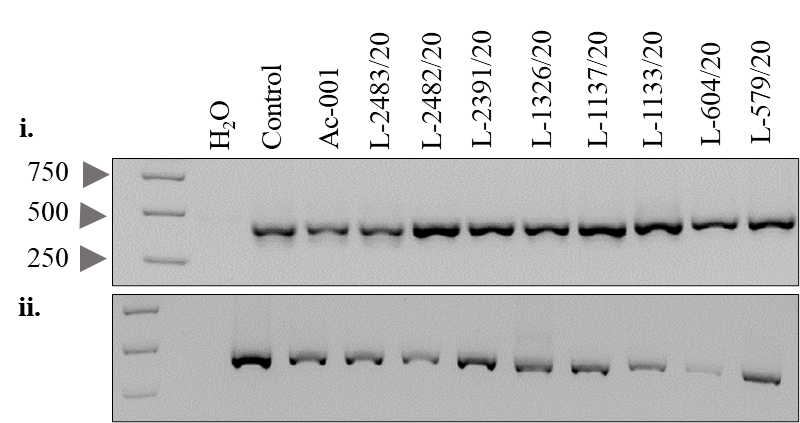 | 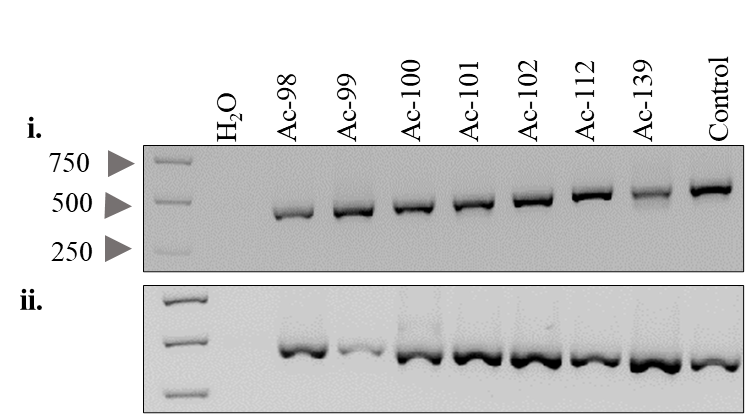 | 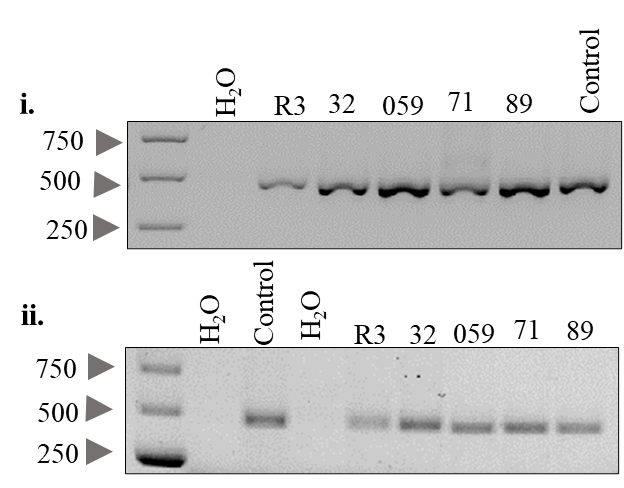 |

**Figure S2:** Agarose gel images depicting PCR amplicons of *Acanthamoeba* isolates (**i**, first panel in each image) recovered from Indian corneal samples along with ATCC-30171 (Ac-001) (**A**), Australian corneal (**B**), and water samples (**C**) and associated intracellular bacteria (**ii**, second panel in each image). Bands were visualised using 2% gel electrophoresis; primer set JDPFw/Rv (18S rRNA) and 341fw/785rv (16S rRNA) yielded 450 bp and 464 bp amplicons, respectively. Positive controls: *A. castellanii* (ATCC 30868) and *E. coli* (ATCC 10798) for 18S rRNA and 16S rRNA PCR assays; and molecular grade water for negative control.





**Figure S3:** Phylogenetic tree of 21 *Acanthamoeba* isolates included in microbiome study based on the partial sequence of the *Rns* gene. The tree was created using the neighbour-joining approach with the Kimura 2-parameter based on 1,000 bootstrap values in MEGA-11. The tree was visualised using the interactive tree of life (iTOLv6). The isolates of this study (green – Indian corneal isolates, ocean blue = Australian corneal isolates, purple = Australian water isolates, yellow = ATCC strain, and sky blue = NCBI reference strains) formed four major genotypic clusters; T4 was the most common genotype (four sub-genotypes: T4A, T4B, T4D, and T4F) and T12, T10, and T5 each had only one isolate.

**Table S3:** 16S V1-3 read statistics after removing low abundant and the non-bacterial ASVs. Note: There was only one ASV (feature ID: 05fda7c7a4dc82cc555d60ca49d81946) that aligned poorly with PICRUSt2 reference data and thus could not be assigned an NSTI value. All other ASVs were assigned NSTI values <2.

| **Features** | **Samples (*n*=20)** |
| --- | --- |
| Total number of samples | 20 |
| MiSeq sequencing reads: |  |
| Maximum read | 132,158 |
| Minimum read | 12,189 |
| Mean read ± SD | 82,549 ± 30,667 |
| Median read | 80,667 |
| Total number of reads | 1,650,988 |
| Number of ASVs | 382 |


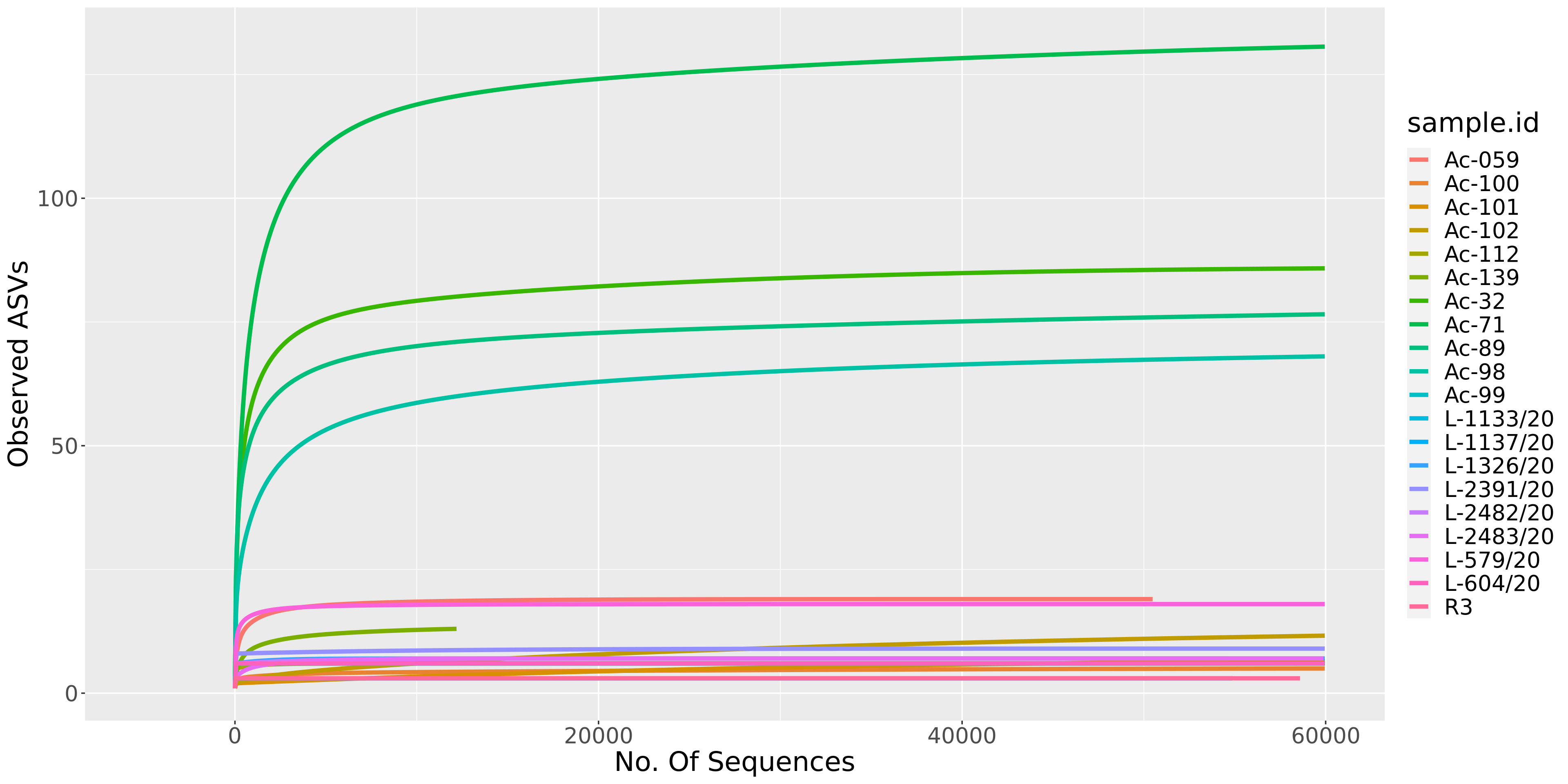


**Figure S4:** Alpha rarefaction curves of observed ASVs for individual *Acanthamoeba* isolates plotted as a function of sequencing depth. The R-package vegan (v2.6.4) was used to generate rarefaction curves with the ggplot function in order to assess whether all diversity of the bacterial community was captured. Each curve represents a sample, which illustrates the number of ASVs identified in a random subset of different numbers of sequencing reads.

| **A** | **B** | **C** |
| --- | --- | --- |
| 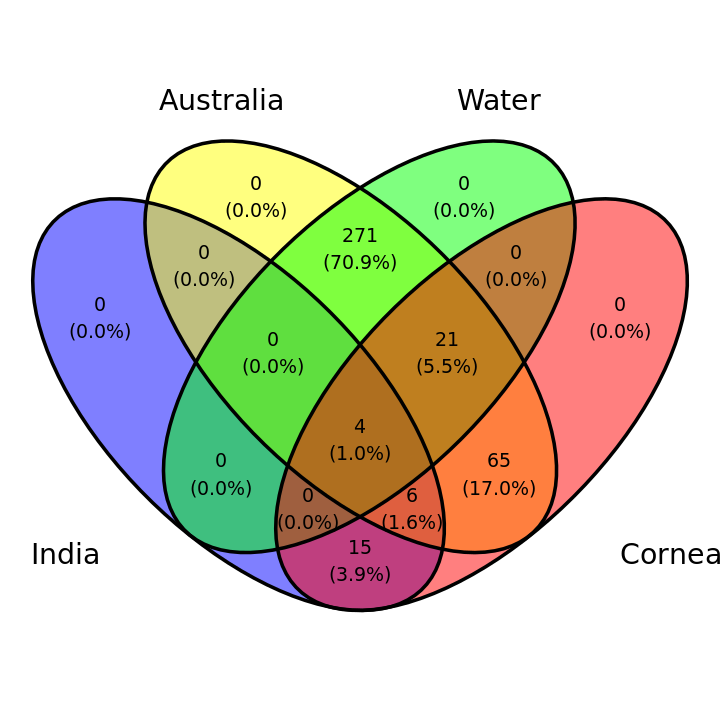 | 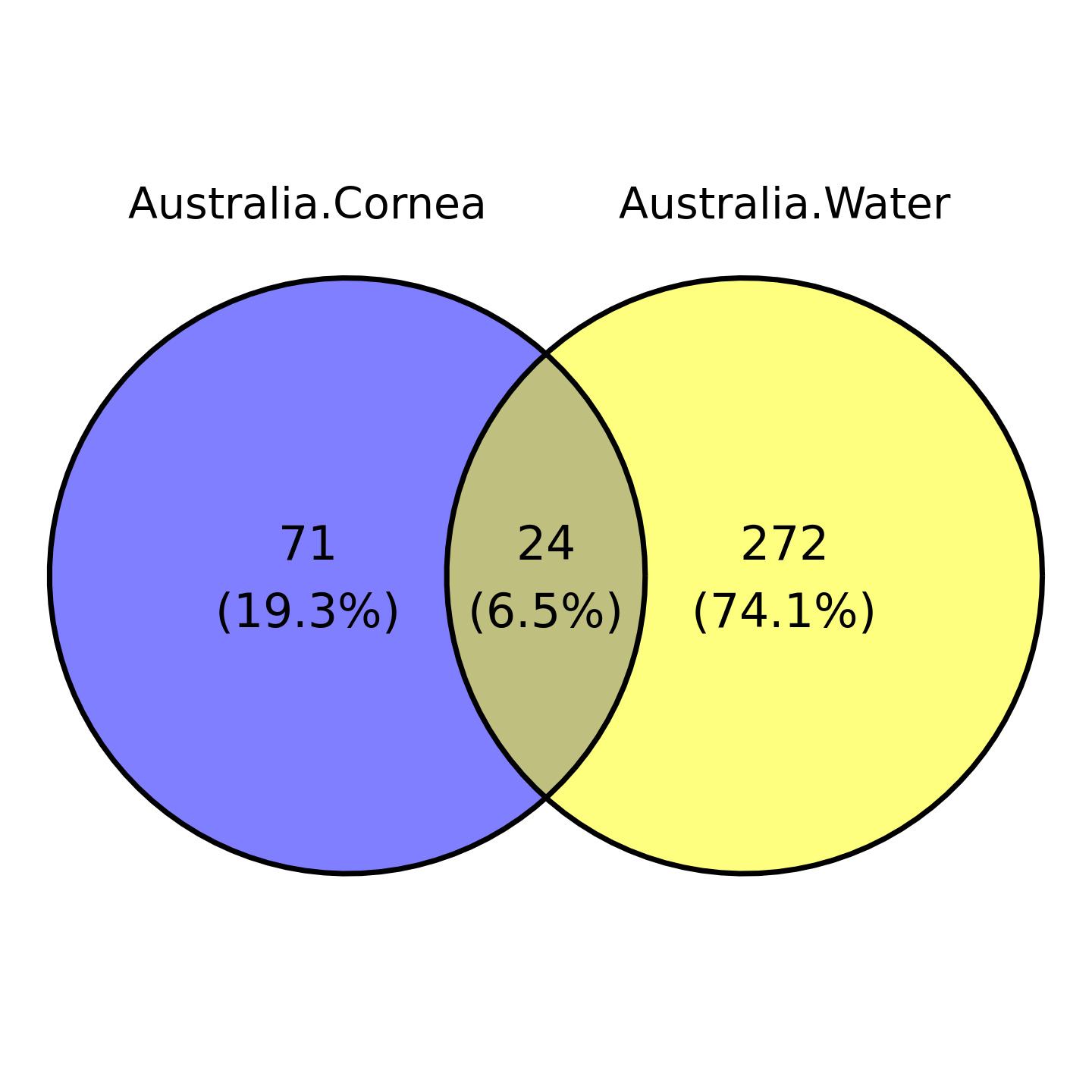 | 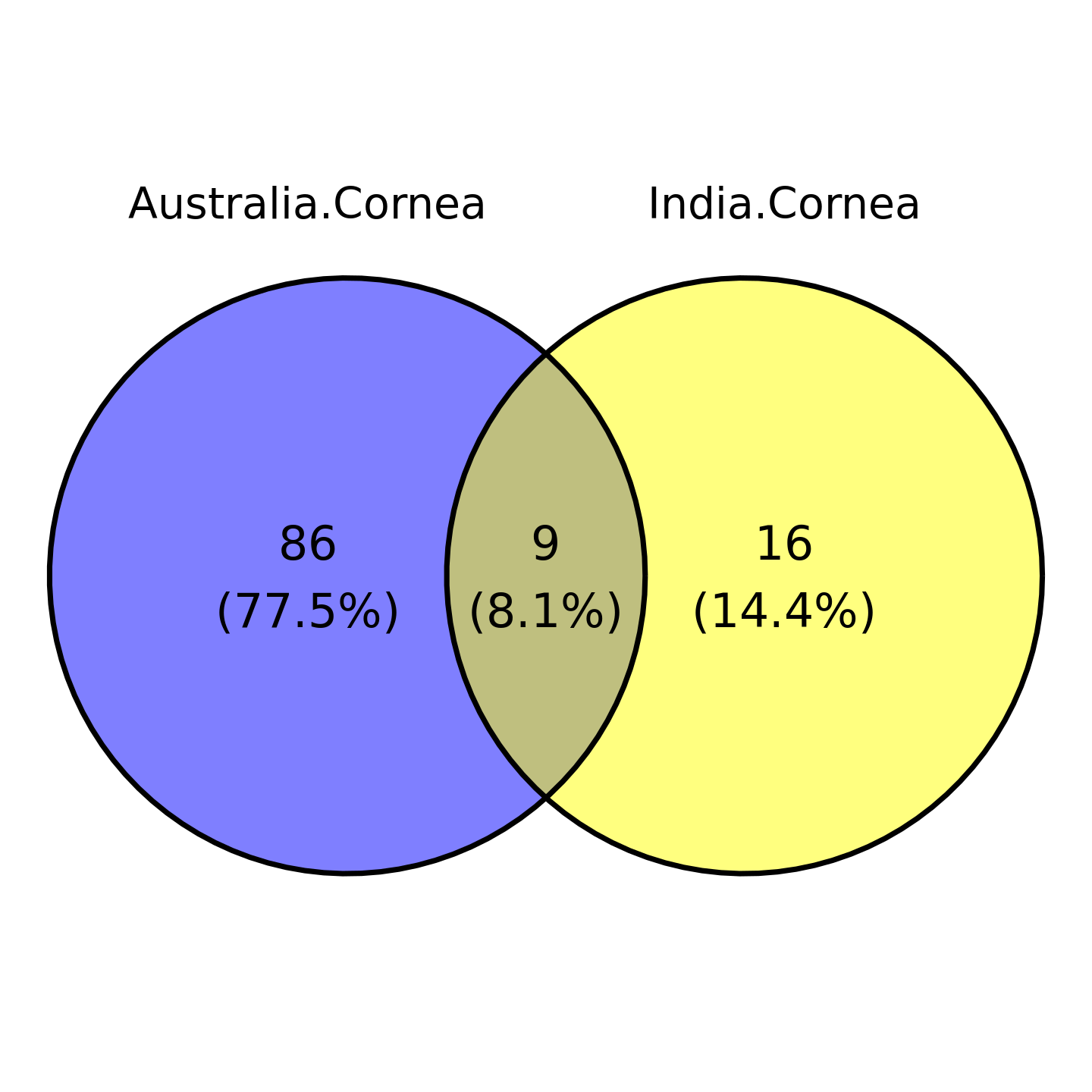 |
| **Figure S5:** Venn diagram representation of the ASVs from the different *Acanthamoeba* groups, (**A**) The unique and shared ASVs between *Acanthamoeba* isolates from cornea and water samples in Australia and corneal specimens in India, (**B**) Between Australian isolates (water and cornea), and (**C**) Between Indiana and Australian corneal isolates. | | |


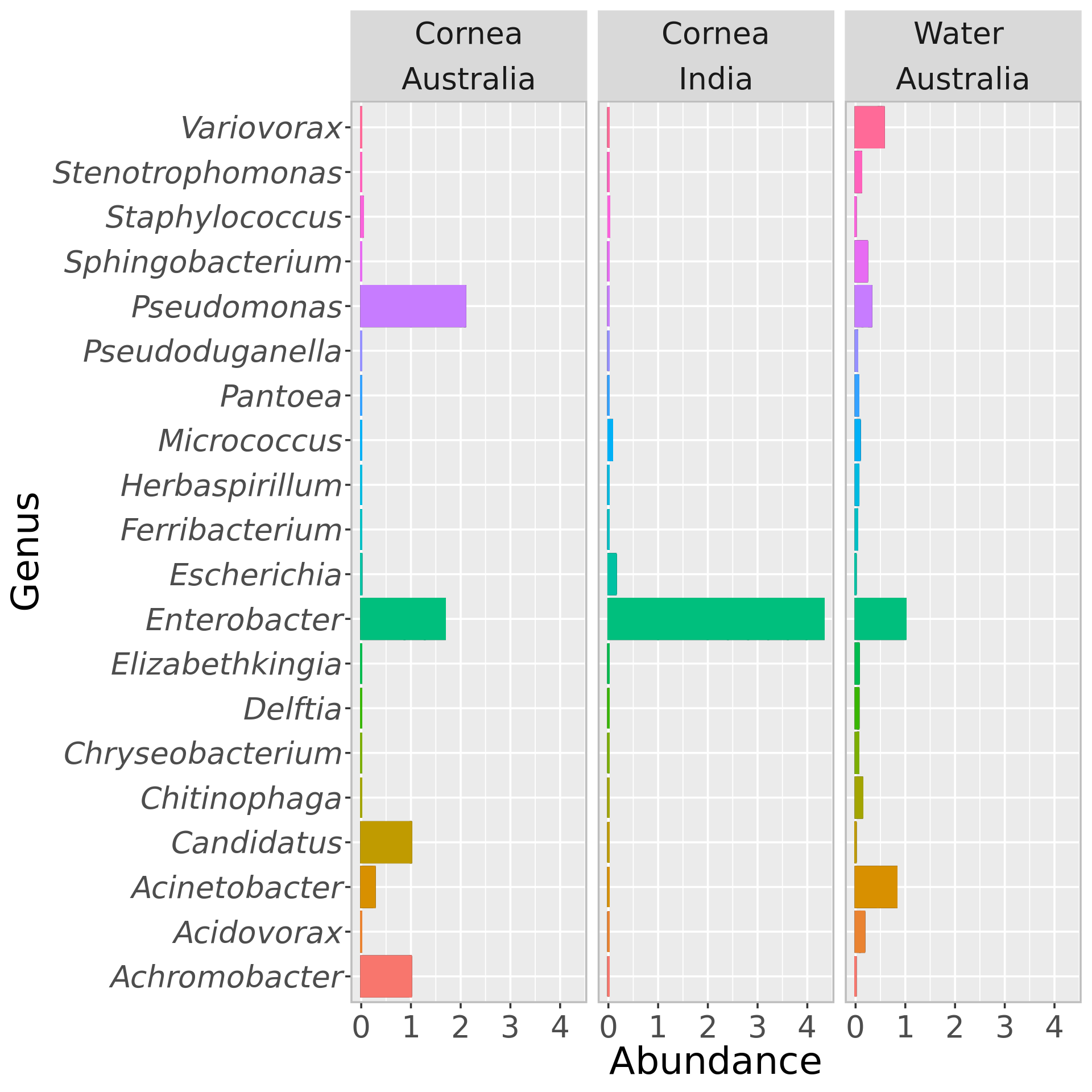


**Figure S6:** The top twenty bacterial genus cross all *Acanthamoeba* isolates as per source of isolation and origin of country. For visualization, ‘*Candidatus*’ was labelled for *Candidatus* Jidaibacter acanthamoeba.


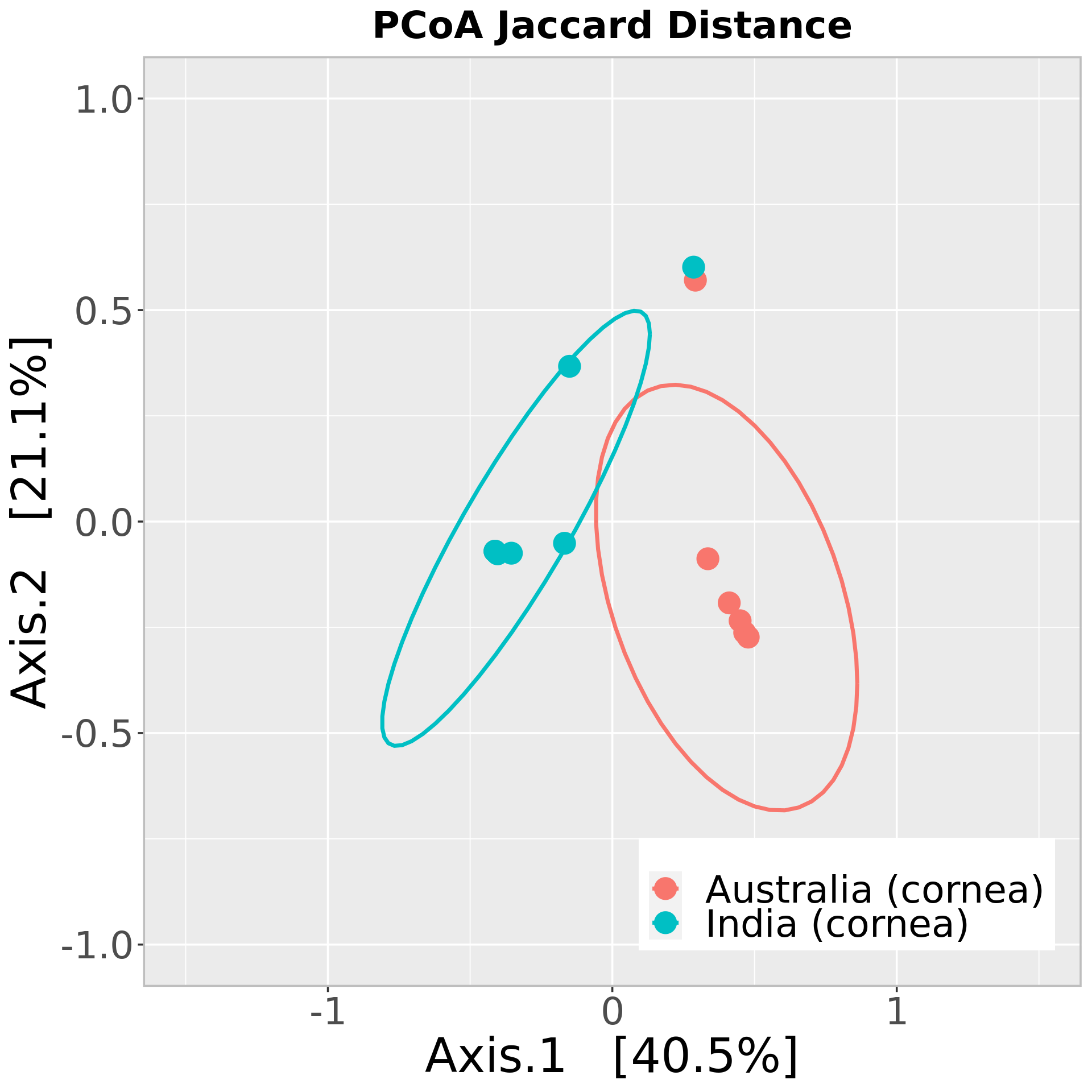


**Figure S7:** Principal coordinates analysis (PCoA) plot using the Jaccard distance index from corneal isolates of Australian (*n* = 7; orange sphere) and Indian (*n* = 8; blue sphere) *Acanthamoeba* isolates. Axes represent the first two principal components of the PCoA, and each point on the plot represents the intracellular bacterial microbiome of an individual *Acanthamoeba* strain (*p*<0.05). The ASVs data were transformed to relative abundance before plotting to account for differences in sequencing depth and some of the sample points are overlapped on the plots due to the very similar bacterial microbiome composition.


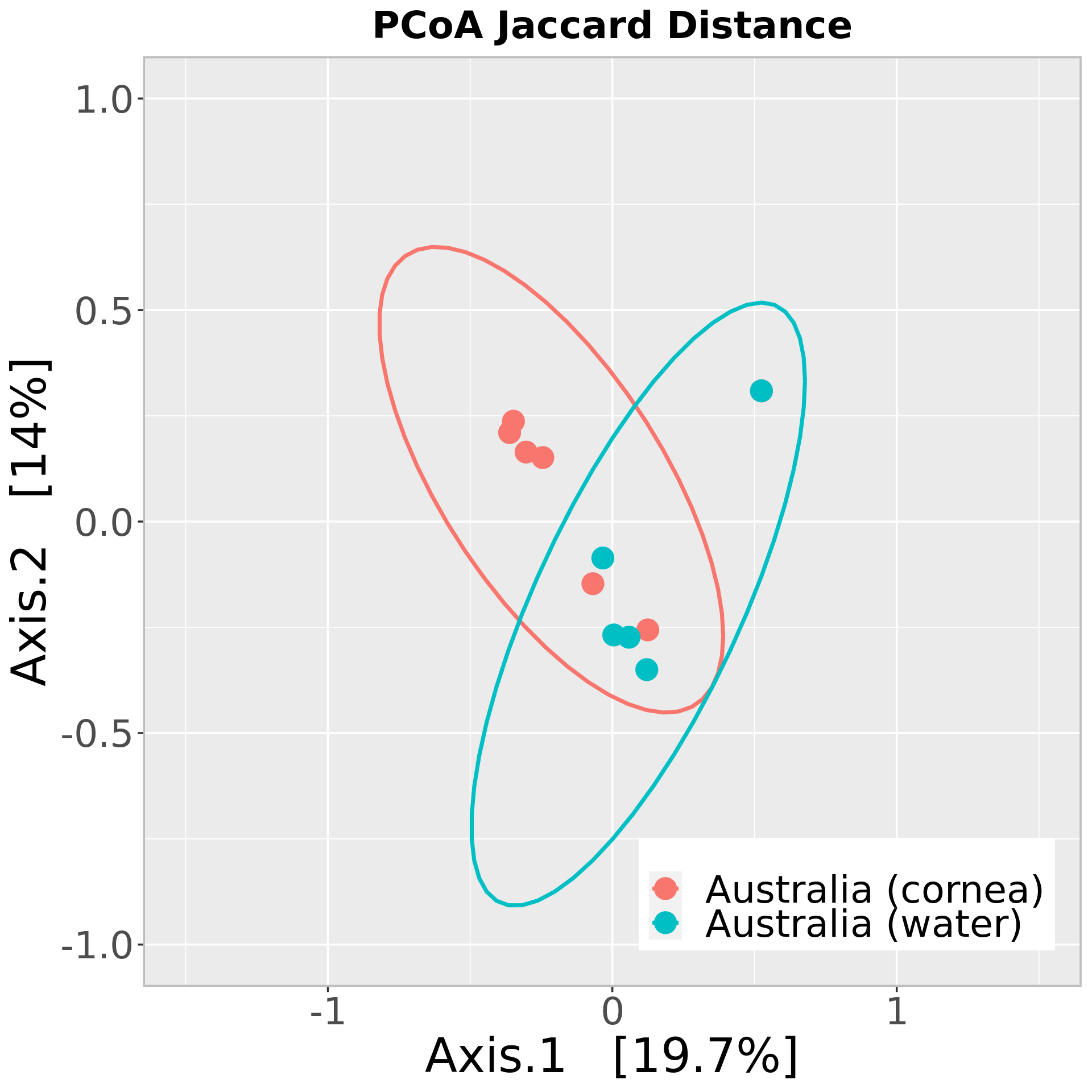


**Figure S8:** Principal coordinates analysis (PCoA) plot using the Jaccard distance index from corneal (*n* = 7; orange sphere) and water isolates (*n* = 5)of Australian *Acanthamoeba* isolates. Axes represent the first two principal components of the PCoA, and each point on the plot represents the intracellular bacterial microbiome of an individual *Acanthamoeba* strain (*p*>0.05). The ASVs data were transformed to relative abundance before plotting to account for differences in sequencing depth and some of the sample points are overlapped on the plots due to the very similar bacterial microbiome composition.





**Figure S9:** Phylogenetic tree of top 21 ASVs identified upto genus level on the partial sequence (V1-3) of 16S rRNA. The tree was created using the neighbour-joining approach with the Kimura 2-parameter based on 1,000 bootstrap values in MEGA-11. The tree was visualised using the interactive tree of life (iTOLv6). The label at genus level (except *Pseudomonas* *putida*) is mentioned with continuous number such as *Escherichia* -1, - 2, - 3, - 4, and - 5. In cases where the genus level classification was not possible, a higher taxonomic level is cited. For visualization, ‘*Candidatus*’ was labelled for *Candidatus* Jidaibacter acanthamoeba.

| **i** | **ii** | **iii** |
| --- | --- | --- |
| 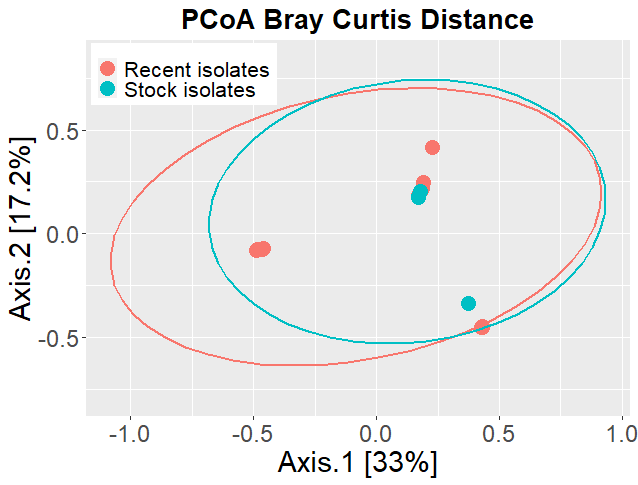 | 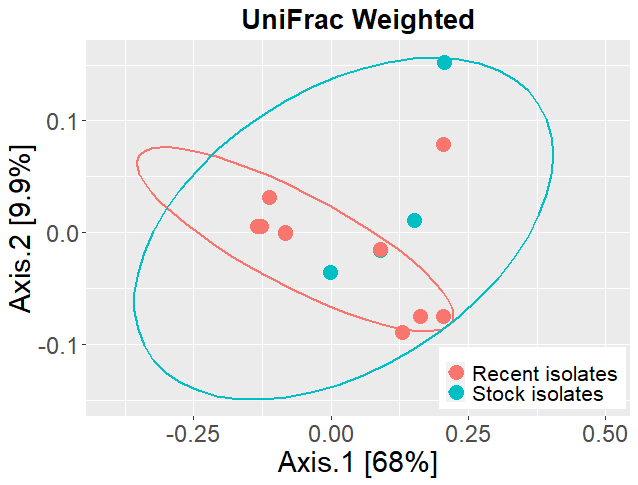 | 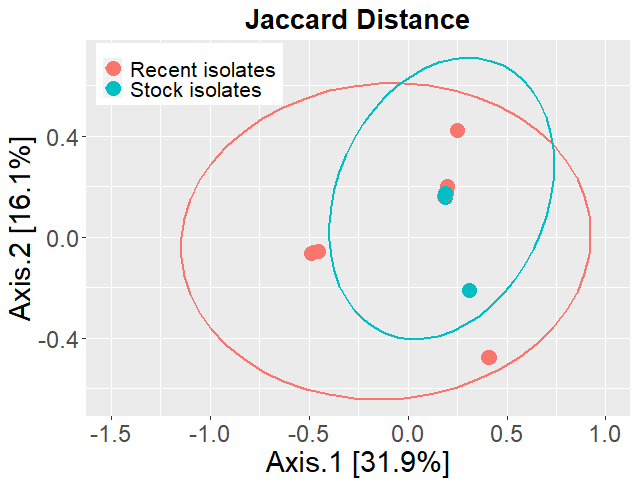 |

**Figure S10:** Intracellular bacterial beta diversity of recent (*n*=14) and stock (*n*=6) isolates of *Acanthamoeba* spp. Two-dimensional PCoA plots for Bray-Curtis dissimilarity index (*p*=0.37) (**i**), weighted distance metric (*p*=0.36) (**ii**), and Jaccard distance index (*p*=0.58) (**iii**) between stock and recent isolates of *Acanthamoeba* spp. (all *p*-values>0.05). Axes represent the first two principal coordinates of the PCoA plot, and each point on the plot represents bacterial microbiome of an individual *Acanthamoeba* isolate (orange = recent, blue = stock). Each plot represents relative abundance of ASVs and some of the sample points are overlapped on the plots due to the very similar bacterial microbiome communities.

| **i** | **ii** |
| --- | --- |
| 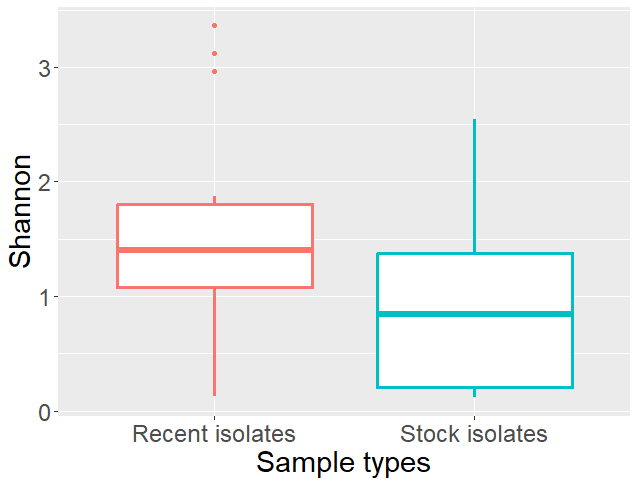 | 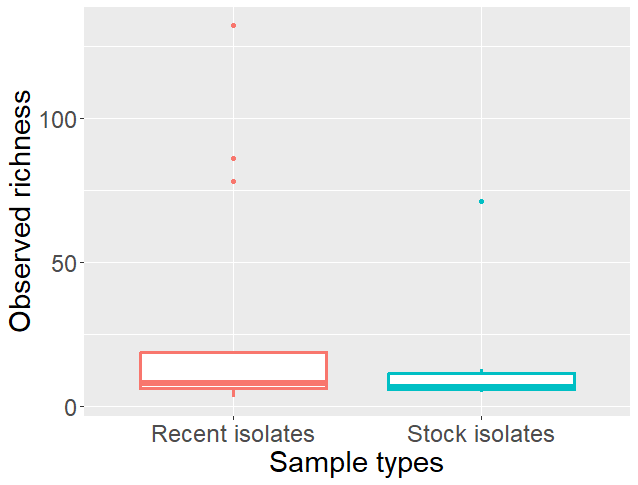 |

**Figure S11:** Alpha diversity of bacterial microbiome composition of recent (*n*=14) and stock (*n*=6) *Acanthamoeba* isolates as measured by Shannon index (*p*=0.1) (**i**), and number of observed ASVs (*p*=0.4) (**ii**). Wilcoxon rank sum test was performed to compare the diversity of two groups. The boxplots show the smallest and largest values (the 25th and 75th quartiles), the median, and outliers.
